# Active Surveillance of Candidemia, Australia

**DOI:** 10.3201/eid1210.060389

**Published:** 2006-10

**Authors:** Sharon Chen, Monica Slavin, Quoc Nguyen, Deborah Marriott, E. Geoffrey Playford, David Ellis, Tania Sorrell, the Australian Candidemia Study

**Affiliations:** * Westmead Hospital, Westmead, New South Wales, Australia;; † Royal Melbourne Hospital, Melbourne, Victoria, Australia;; ‡ St. Vincent's Hospital, Sydney, New South Wales, Australia;; § Princess Alexandra Hospital, Brisbane, Queensland, Australia;; ¶ Women's and Children's Hospital, Adelaide, South Australia, Australia;; # University of Sydney, Sydney, New South Wales, Australia

**Keywords:** Population-based, candidemia, surveillance, epidemiology, Australia, healthcare-associated, Candida, research

## Abstract

Population-based surveillance for candidemia in Australia from 2001 to 2004 identified 1,095 cases. Annual overall and hospital-specific incidences were 1.81/100,000 and 0.21/1,000 separations (completed admissions), respectively. Predisposing factors included malignancy (32.1%), indwelling vascular catheters (72.6%), use of antimicrobial agents (77%), and surgery (37.1%). Of 919 episodes, 81.5% were inpatient healthcare associated (IHCA), 11.6% were outpatient healthcare associated (OHCA), and 6.9% were community acquired (CA). Concomitant illnesses and risk factors were similar in IHCA and OHCA candidemia. IHCA candidemia was associated with sepsis at diagnosis (p<0.001), death <30 days after infection (p<0.001), and prolonged hospital admission (p<0.001). Non–*Candida albicans* species (52.7%) caused 60.5% of cases acquired outside hospitals and 49.9% of IHCA candidemia (p = 0.02). The 30-day death rate was 27.7% in those >65 years of age. Adult critical care stay, sepsis syndrome, and corticosteroid therapy were associated with the greatest risk for death. Systematic epidemiologic studies that use standardized definitions for IHCA, OHCA, and CA candidemia are indicated.

## Abstract 2

This infection has a high death rate and is predominantly associated with healthcare.

Bloodstream infections with *Candida* (candidemia) account for 8% to 15% of hospital-acquired sepsis in the United States (*1**,**2*). Death rates associated with candidemia are high (40%–70%) (*3*), with an estimated attributable death rate of 25% to 49% (*4**,**5*). Candidemia also results in prolonged hospital stay and substantial healthcare costs (*4**,**6*).

Studies have shown that incidence and etiology of hospital-acquired candidemia varied with geography, type of hospital, population studied, and clinical practice (*3**,**7**–**9*). Secular trends in the incidence of candidemia and etiologic species of *Candida* differ between Europe and the United States (*10*). Although candidemia is perceived as a hospital-acquired infection, changes in medical practice to more frequent use of home healthcare for many illnesses and long-term indwelling vascular devices have increased the number of susceptible patients in the community, potentially increasing incidence and changing epidemiology.

Two population-based surveys in the United States showed that 20% and 28% of cases were acquired outside hospitals (*7**,**11*), compared with only 1.2% in Finland (*12*). In a more recent study in Spain, 11% of episodes occurred in outpatients (*13*). These studies are not directly comparable because definitions of outpatient-acquired episodes were different. In addition, within this group, little distinction was made between cases of healthcare-associated candidemia and community-acquired (CA) candidemia.

A retrospective survey of candidemia in Australian hospitals from 1995 to 1998 showed an increase in the annual incidence of infection and a decrease in the proportion caused by *Candida albicans* (*14*). The Australian Candidemia Study Group was formed in 2001 to conduct the first countrywide, population-based, active laboratory surveillance for candidemia.

## Methods

### Study Design and Data Collection

Cases of candidemia were prospectively identified by blood culture surveillance at 50 of 52 public and private microbiology laboratories in Australia (population ≈20.1 million) from August 2001 to July 2004. One nonparticipating laboratory provided services to a university pediatric hospital where candidemia rarely occurred (A. Daley, pers. comm.) and the other provided services to an adult university hospital (36 cases identified; H. Sheorey, pers. comm.). Approval for the study was obtained from the human ethics review committees of institutions providing clinical data.

Adults, children, and neonates with <1 blood culture yielding *Candida* species were eligible for enrollment. State or territory coordinators were responsible for case identification. Information was collected on a standard form regarding age, sex, patient location at time of candidemia diagnosis, healthcare setting, risk factors within the preceding 30 days (including surgery, vascular access devices [VADs], hyperalimentation, and use of antimicrobial and systemic antifungal agents), major concomitant conditions (International Statistical Classification of Diseases and Related Health Problems, 10th revision, Australian modification) (*15*), portal of entry, clinical signs of sepsis (*16*), complications of candidemia, results of diagnostic studies, antifungal therapy, and clinical outcome <30 days after diagnosis. All data were collected prospectively, and forms were completed on days 5 and 30 after the date of the initial positive blood culture or at death if it occurred earlier. Data were collected and analyzed at a central site. Periodic audits of laboratory records ensured that all cases of candidemia were reported. For the study period, number of hospital beds and annual different day patient separations (defined as completed admissions) were obtained from 40 hospitals.

### Definitions

A case was defined as incident isolation of *Candida* species from blood during the study period. For patients with >1 episode of candidemia, the second episode was defined as a new case if it occurred >30 days after the previous episode. A total of >2 episodes fulfilling the case definition and occurring in different patients who were epidemiologically linked (e.g., with regard to risk factors, location, and time) constituted a case cluster.

Episodes were classified as inpatient healthcare-associated (IHCA) if they occurred >48 hours after hospital admission and had not clinically manifested on admission (*17*). Cases occurring <48 hours after hospital admission were considered outpatient-acquired. Among outpatient-acquired candidemia, episodes associated with an indwelling medical device, surgical procedure, or chemotherapy-related neutropenia (<1 × 10^9^ cells/L, adjusted for children) were classified as outpatient healthcare associated (OHCA), and CA infections were classified as those occurring in patients with no healthcare-related risk factors (*17*). Adult intensive care unit (ICU) acquisition was when candidemia developed >48 hours after ICU admission for nonneutropenic patients. Source of candidemia was considered VAD related if culture of the device tip grew the same species isolated from blood. Endocarditis was classified by modified Duke criteria (*18*).

### Microbiologic Methods

All laboratories cultured blood specimens in BACTEC (Becton Dickinson, Sparks, MD, USA) or BacT/Alert 3D (bioMérieux, Marcy l'Etoile, France) automated systems. *Candida* organisms were speciated by using standard phenotypic methods (*19*). Isolates were forwarded to a reference laboratory (Women's and Children's Hospital, Adelaide) for susceptibility testing and species confirmation by using conventional methods (*20*). *Candida dubliniensis* was distinguished from *C*. *albicans* by PCR fingerprinting (*21*). Where identification was discordant, species determination at the reference laboratory was used.

### Statistical Analysis

Population and age-specific incidences were calculated by using denominator data from the 2004 Australian census (*22*) and hospital-specific incidences by using day of separation denominator data from individual hospitals for the study period. Overall incidences were expressed as pooled mean rates calculated by aggregating numerators and dividing by the sum of denominators from all sites. Data were analyzed with SPSS version 10.0.7 (SPSS Inc., Chicago, IL, USA). Continuous variables were compared with Student *t* test, and categorical variables were compared with χ^2^ or Fisher exact tests. Incidence data from university hospitals were pooled and compared with pooled data from other hospital types with χ^2^ test. A p value <0.05 was considered significant. Univariate analyses were performed to identify risk factors associated with overall illness. Candidate variables with a univariate p<0.15, or previously published risk factors, were analyzed by multiple logistic regression. The same independent predictors of death were identified with forward, backward, and stepwise variable selection methods.

## Results

### Incidence and Patient Demographics

Permission was denied to include 36 candidemia episodes identified at the nonparticipating adult hospital. Thus, the number of incident episodes was 1,095 (in 1,095 patients), with 337, 352, and 406 during the first, second, and third years, respectively, of the study. Data on hospital characteristics were available for 1,095 patients, demographic and clinical data for 1,005 (91.7%) episodes, and outcome data for 857 (78.3%). Species were identified for 1,068 (97.5%) isolates.

**Figure 1:**
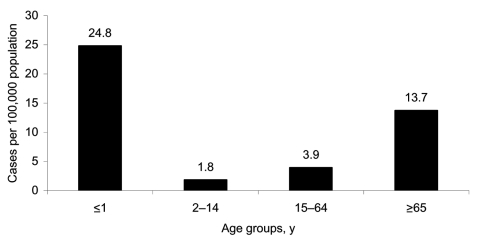
Annual age-specific incidence of candidemia, Australia, 2001–2004.

The average population-based incidence of candidemia was 1.81/100,000 (1.87/100,000 if inclusive of cases at the nonparticipant hospital) per year (Table 1) and was highest in infants (24.8/100,000) and persons >65 years of age (13.7/100,000; Figure 1). Most cases (36.4%) occurred in the most populous state, New South Wales, but incidence was highest in Queensland. Age composition of the population was similar by jurisdiction and similar to the national average (data not shown) except for the 2 least populated areas, Northern Territory and Australian Capital Territory, where the percentage of persons >65 years of age was 4.4% and 9.3%, respectively, compared with the national average of 13%.

**Table 1:** Number and incidence of candidemia cases reported in all jurisdictions, Australia, 2001–2004*

Parameter	State or territory								
	NSW	VIC	QLD	SA	WA	TAS	NT	ACT	Total
No. cases (%)	399 (36.4)	300 (27.4)	273 (24.9)	45 (4.1)	40 (3.7)	19 (1.7)	5 (0.5)	14 (1.3)	1,095 (100.0)
Mean incidence per 100,000 population	1.98	2.01†	2.34	0.99	0.67	1.31	0.83	1.44	1.81†
Mean incidence per 1,000 separations‡	0.19	0.36	0.18	0.25	0.16	0.09	0.05	0.09	0.21
No. institutions	35	15	30	3	3	2	1	1	90
Acquisition (% of episodes)§									
IHCA	79.1	83.4	79.3	90.2	87.5	89.5	50	85.7	81.5
OHCA	10.9	14.2	10.7	9.8	2.5	10.5	0	14.3	11.6
CA	10.0	2.4	10.0	0	10.0	0	50.0	0	6.9

*NSW, New South Wales; VIC, Victoria; QLD, Queensland; SA, South Australia; WA, Western Australia; TAS, Tasmania, NT, Northern Territory; ACT, Australian Capital Territory; IHCA, inpatient healthcare-associated; OHCA, outpatient healthcare-associated; CA, community-acquired. †Mean incidences per 100,000 in VIC and in Australia are 2.25 and 1.87, respectively, with inclusion of 36 cases identified at the nonparticipating adult hospital. ‡Data available for 17 hospitals in NSW, 8 in VIC, 8 in QLD, 2 in SA, 1 in WA, 2 in TAS, 1 in NT, and 1 in ACT. §Data for 919 candidemia episodes.

The pooled mean annual hospital-specific incidence of candidemia (data from 40 institutions) was 0.21/1,000 separations (range by jurisdiction 0.05–0.36) (Table 1). No case clusters occurred. The estimated mean incidence in university (n = 28) and university-affiliated hospitals (n = 9) was similar (0.22/1,000 separations, range by jurisdiction 0.05–0.90) and greater than in private hospitals (n = 3, 0.1/1,000 separations, range by jurisdiction 0.04–0.16).

Neonates <1 month of age accounted for 33 (3.3%) of 1,005 cases, children 2 months to 14 years of age for 95 (9.5%) cases, adults 15–64 years of age for 527 (52.4%) cases, and patients >65 years of age for 350 (34.8%) cases. The median age was 56 years (range 0–98); 537 (53.4%) episodes affected male patients.

Most cases occurred in medical wards (including cancer and hemopoietic stem cell transplant [HSCT] units; 390 [35.6%] of 1,095 cases), critical care units (273 [24.9%] cases), and surgical wards (193 [17.3%] cases). Emergency (4.3%) and obstetrics and gynecology (0.5%) services reported small percentages of episodes. Pediatric cases occurred in pediatric critical care units (16 cases, 1.5%) and pediatric medical or surgical wards (24 cases, 2.2%). Neonatal infections occurred in premature or low birthweight infants in neonatal critical care units (35 cases, 3.2%).

### Concomitant Conditions, Risk Factors, and Healthcare Settings for Candidemia

Concomitant conditions and risk factor data were available for 1,005 episodes. Cancer was the most common underlying condition (323 cases, 32.1%), with 164 episodes in solid tumor (50.8% cancers) cancer patients. Of 159 cases diagnosed in patients with hematologic malignancy, 91 occurred in patients with leukemia and 58 in those with lymphoma. Only 52 episodes occurred in transplant recipients (17 allogeneic and HSCTs, 11 autologous HSCTs, and 24 solid organ transplants). Gastrointestinal disorders (19%), chronic cardiovascular disease (13.8%), and diabetes (13.6%) were common, but pancreatitis was infrequent (2.5%), and coincident HIV infection was rare (0.6%). Common iatrogenic risk factors included indwelling VADs (72.6%), antimicrobial agents (77%), major surgery (37.1%), and hyperalimentation (33%, Table 2).

**Table 2:** Selected concomitant conditions, risk factors, and outcomes for 919 episodes of candidemia by healthcare setting, Australia, 2001–2004*

Characteristic		Healthcare-associated, outpatient acquired (n = 107)	Healthcare-associated, inpatient acquired (n = 749)	p value†	Community acquired (n = 63)	p value†
Concomitant condition						
	Hematologic malignancy	27 (25.2)	131 (17.5)	0.06	1 (1.6)	<0.001
	Solid organ malignancy	21 (19.6)	135 (18.1)	0.69	8 (12.7)	0.30
	Solid organ transplantation	2 (1.9)	22 (2.9)	0.76	–	–
	HSCT	3 (2.8)	25 (3.4)	1.0	–	–
	Prematurity	–	35 (4.7)	–	1 (1.6)	0.37
	Renal disease‡	10 (9.3)	56 (7.5)	0.44	2 (3.2)	0.24
	GI and liver disease§	17 (15.9)	162 (21.6)	0.20	12 (19.0)	0.67
	Pancreatitis	1 (0.9)	24 (3.2)	0.35	–	–
	Cardiovascular disease¶	17 (15.9)	118 (15.8)	1.0	4 (6.3)	0.09
	Diabetes mellitus	14 (13.1)	111 (14.8)	0.77	12 (19.0)	0.37
Risk factor						
	Surgery in past 30 d	16 (15.7)	353 (49.0)	<0.001	4 (6.9)	0.08
	Burns/trauma	–	40 (5.5)	–	1 (1.8)	–
	VAD	72 (69.2)	653 (90.8)	<0.001	5 (9.1)	<0.001
	Hyperalimentation	13 (12.6)	318 (44.1)	<0.001	1 (1.8)	0.04
	Neutropenia	19 (18.1)	144 (19.6)	0.79	1 (1.6)	0.002
	Antimicrobial agents	69 (65.7)	686 (95.5)	<0.001	19 (34.5)	<0.001
	Corticosteroids	29 (28.4)	236 (33.0)	0.43	1 (1.6)	<0.001
	Chemotherapy	27 (25.2)	106 (14.2)	0.01	–	–
	Systemic antifungal use	15 (14.0)	106 (14.2)	1.0	–	–
	Intravenous drug use	2 (1.9)	13 (1.7)	1.0	15 (23.3)	<0.001
	Other BSI	15 (14)	256 (34.2)	<0.001	5 (8)	0.33
	Sepsis syndrome	74 (69.2)	594 (79.3)	0.01	37 (58.7)	0.71
	*Candida* endocarditis	7 (6.5)	22 (2.9)	0.08	5 (7.9)	0.55
	Mean time in hospital, d	18.1	56.7	<0.001	16.1	0.33
	Death within 30 d	13 (12.4)	218 (31.1)	<0.001	6 (9.4)	1.0

*Data are no. (%) of total cases in each category, except for mean time in hospital. Some patients had >1 concomitant condition or risk factor. HSCT, hemopoietic stem cell transplant; GI, gastrointestinal; VAD, vascular access device; BSI, bloodstream infection. †By χ^2^ test using outpatient healthcare-associated data as baseline. ‡Hemodialysis or peritoneal dialysis. §Biopsy-proven cirrhosis with portal hypertension, past upper GI bleeding caused by portal hypertension, or prior episodes of hepatic failure. ¶Severe congestive heart failure. Symptoms at rest/inability to carry out physical activity without discomfort.

Of 919 episodes for which information on the healthcare setting was available, 749 (81.5%) were IHCA, 107 (11.6%, or 62.9% of outpatient-acquired cases) were OHCA, and 63 (6.9%) were CA. The proportion of disease acquired outside a hospital by jurisdiction ranged from 9.8% (4 of 41 cases in South Australia) to 50% (but only 1 of 2 cases in Northern Territory). Of the major states, Western Australia reported the lowest percentage of OHCA cases (1 [2.5%] of 40 episodes, Table 1). Comparison of major concomitant conditions and risk factors according to healthcare setting is summarized in Table 2; >4 risk factors were evident in 195 (21.2%) episodes. A total of 183 (20%) episodes were associated with use of an adult ICU. Twenty-nine (93%) neonates received hyperalimentation compared with 35.4% of the adults. Systemic antifungal agents were administered as prophylaxis in 67 (55.4%) of 121 instances; 48 (68.6%) of these prescriptions were for hematology/HSCT patients.

Most (90%–100%) episodes associated with prematurity, organ transplantation, and burn or trauma were IHCA (Table 2). Compared with all cases acquired outside a hospital, patients with IHCA infection were significantly more likely to have had recent surgery (49% vs. 12.5%, p<0.001), neutropenia (19.7% vs. 12.6%, p = 0.04), indwelling VADs (90.1% vs. 48.4%, p<0.001), corticosteroid therapy (33% vs. 19%, p<0.001), or parenteral nutrition (44.1% vs. 8.9%, p<0.001) or to have died within 30 days of infection (31.1% vs. 1.8%, p<0.001). These risk factors, with the exception of surgery, were more common in cases with OHCA candidemia than in those with CA candidemia (Table 2).

Recent surgery and VADs, hyperalimentation, and antimicrobial agents were more common in IHCA patients than in OHCA patients (Table 2). Conversely, coincident malignancy (44% vs. 34% episodes, p = 0.05) and cancer chemotherapy (25.2% vs. 14.3%, p = 0.01) were associated with OHCA infection. OHCA patients also included hemodialysis recipients and patients with diabetes. In CA candidemia, common concomitant conditions included intravenous drug use (IVDU, 23.8%), gastrointestinal disorders (19%), diabetes (19%), behavioral disorders such as chronic alcohol abuse (31.8%), and infectious diseases (23.4%); 15 (50%) of 30 episodes in IVDU patients were CA (Table 2). Differences in clinical manifestations were identified in the 3 groups. Patients with IHCA candidemia were more likely to have sepsis, die within 30 days, remain in hospital longer, and have concomitant healthcare-associated bacteremia (Table 2). Of 34 patients with *Candida* endocarditis (13 definite and 21 possible), 7 were OHCA with an increasing trend when compared with those with IHCA candidemia (Table 2).

### *Candida* Species

Species of *Candida* was correctly identified in 96.7% instances in which identity was established. *C*. *albicans* was the most common species (505 [47.3%] of 1,068 episodes) followed by *C*. *parapsilosis* (19.9%) and *C*. *glabrata* (15.4%). *C*. *tropicalis*, *C*. *krusei*, and *C*. *dubliniensis* were identified in 5.1%, 4.3%, and 1.9%, respectively, of the patients. The remaining 42 (4.0%) episodes were caused by *C*. *guilliermondi* (n = 11), *C*. *lusitaniae* (n = 7), *C*. *kefyr* (n = 5), *C*. *famata* (n = 3), *C*. *rugosa* (n = 3), and *C*. *pelliculosa* (n = 3). There were 24 (2.2%) episodes of polycandidal infection and an increase in the proportion of *C*. *glabrata* candidemia from 10.1% episodes the first year to 19.5% episodes in the third year of the study (p = 0.02, data not shown).

*C*. *albicans* was the predominant species in Tasmania (60%), Australian Capital Territory (77%), and Western Australia (54.1%). However, this species caused only 37.8% of episodes in South Australia, whereas 31.1% were caused by *C*. *parapsilosis* (7.7%–21.9% of cases elsewhere). *C*. *krusei* and *C*. *dubliniensis* candidemia were rare in the least populous jurisdictions where complex procedures such as HSCT are not performed. Age distribution, including proportion of neonates and children in the population, was comparable for all jurisdictions (data not shown).

Species other than *C*. *albicans* were isolated more often from patients with candidemia acquired outside a hospital (60.5% vs. 49.9% for IHCA candidemia, p = 0.02). *C*. *parapsilosis* was recovered from 30.9% of these episodes (compared with 16.7% of IHCA episodes, p<0.001), although *C*. *krusei* and *C*. *dubliniensis* candidemia was rare. Relative proportions of infections caused by *C*. *albicans* and non–*C*. *albicans* species in cases of OHCA candidemia and CA candidemia were similar.

**Figure 2:**
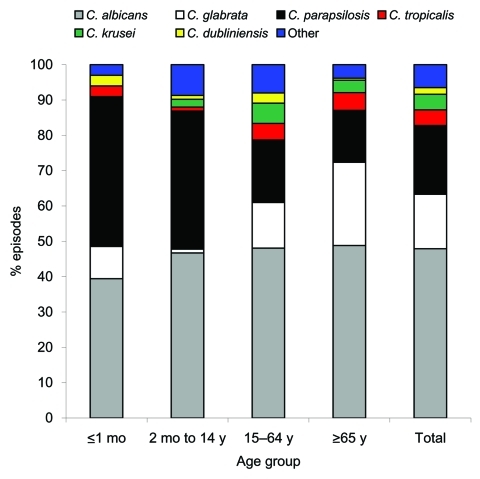
Distribution of causative pathogen according to patient age for 978 *Candida* species, Australia, 2001–2004.

In neonates and children, *C*. *parapsilosis* accounted for a similar proportion of episodes as did *C*. *albicans*; other species were rare. *C*. *albicans* was the most common causative pathogen in adults (48.6% of cases) and *C*. *glabrata*, *C*. *parapsilosis*, and other *Candida* species were approximately equally distributed (Figure 2). Eighty (53.3%) of 150 episodes in patients >65 years of age were caused by *C*. *glabrata*.

*C*. *albicans* was the most common pathogen in ICUs (61.7% of episodes) and surgical (54.4% of episodes) patients but caused only 32% of episodes in hematology patients, who had the highest proportion (24 [66.7%] of 36 episodes) of infection with *C*. *krusei*. *C*. *parapsilosis* accounted for 42.9% of episodes in neonatal ICUs and for 42.4% of cases in patients without hematologic cancer. *C*. *glabrata* was present in all patient groups. Twelve (63.2%) of 19 episodes of *C*. *dubliniensis* candidemia were in surgical and nonhematology patients.

### Outcome

The all-cause 30-day death rate was 27.7% (236 of 853 episodes) and similar in all age groups. Clinician-reported attributable deaths were lower (93 episodes, 10.9%). The death rate was higher in IHCA episodes than in either OHCA or CA episodes (Table 2). Univariate analysis showed that numerous variables were associated with increased death (Table 3). Multiple logistic regression analysis of 16 variables showed that an age >65 years, ICU stay, sepsis at diagnosis, and corticosteroid therapy (p<0.001, Table 4) were associated with the greatest risk for death. *C*. *glabrata* infection was also an independent predictor of death. Treatment with an antifungal agent (752 [85.8%] of 876 episodes) was independently associated with lower odds of death but removal of a VAD (76.4% of 878 instances where VADs were in situ at diagnosis) was not (Table 4). However, in the subset of VAD-related candidemia episodes (n = 409), removal of the VAD (n = 339) was associated with a better outcome (odds ratio 0.48, 95% confidence interval 0.26–0.88, p = 0.02).

**Table 3:** Univariate predictors of death by candidemia 30 days after diagnosis, Australia, 2001–2004*

Variable	Deaths, % (no./total)	Nondeaths, % (no./total)	p value
Age >65 y	53.8 (127/236)	28.4 (175/617)	<0.001
Malignancy	36 (85/236)	33.2 (205/617)	0.44
Hematologic malignancy	16.9 (40/236)	16.5 (102/617)	0.88
Lymphoma	8.1 (19/236)	5.0 (31/617)	0.09
Surgery in past 30 days	44.4 (104/234)	41.2 (254/616)	0.40
VAD	87.1 (203/233)	82.2 (505/614)	0.09
Hyperalimentation	44.2 (103/233)	35.3 (217/615)	0.02
Hemodialysis	16.7 (39/234)	8.3 (51/615)	<0. 001
Urinary catheter/drainage device	69.2 (162/234)	53 (325/613)	<0.001
Trauma/burns	1.7 (4/234)	5.8 (36/616)	0.01
Corticosteroid therapy	44.9 (105/234)	25.6 (158/616)	<0.001
Antimicrobial drug use	96.1 (224/233)	85.5 (526/615)	<0.001
Neutropenia	20.9 (49/234)	16.4 (101/614)	0.126
ICU stay	34.3 (81/236)	15.9 (98/617)	<0.001
Sepsis present (day 0)	87.6 (205/234)	78.8 (484/614)	0.003
Treatment with antifungal agent†	70.5 (165/234)	91.7 (564/615)	<0.001
VAD removal	61.6 (98/159)	82.5 (406/492)	<0.001
*Candida glabrata* infection	22.5 (53/236)	12.6 (78/617)	<0.001
Polycandidal infection	3.8 (9/236)	1.8 (11/617)	0.08
Inpatient healthcare-associated	91.9 (218/237)	85.7 (531/620)	0.02

*VAD, vascular access device; ICU, intensive care unit. †Includes all patients receiving >1 therapeutic dose of systemic antifungal agent(s) as advised by an infectious diseases physician.

**Table 4:** Multivariate predictors of death by candidemia 30 days after diagnosis, Australia, 2001–2004*

Characteristic	OR	95% CI	p value
Age >65 y	3.0	2.0–4.3	<0.001
ICU stay	3.2	2.1–5.0	<0.001
Corticosteroid therapy	2.8	1.9–4.1	<0.001
Hemodialysis	2.4	1.4–4.1	0.002
Hyperalimentation	1.7	1.1–2.5	0.006
Antimicrobial drug use	2.1	0.9–4.4	0.07
Neutropenia	2.2	1.3–3.6	0.002
Sepsis present	3.1	1.7–5.4	<0.001
Treatment with antifungal agent	0.01	0.06–0.2	<0.001
*Candida glabrata* infection	1.8	1.1–2.9	0.01

*Sixteen candidate variables were included in the final model. OR, odds ratio; CI, confidence interval; ICU, intensive care unit.

## Discussion

This study provides the first contemporary, comprehensive, population-based description of candidemia across a continent. Overall disease incidence (1.81/100,000) was similar to that in Europe (1.4–4.9/100,000) (*12**,**23*) but different from that in the United States (6–10/100,000) (*7**,**11**,**24*). Most US and European surveys were not population-based and those that were included patients from specific areas (*11**,**13**,**24*). Age distribution of the population affected estimates, as shown by higher incidence among young and elderly people in Australia. Geographic variations in incidence were observed across Australia. However, this finding was unlikely to be caused by differences in age distribution because this distribution was similar in all states. Although the proportion of elderly persons was lower in Northern Territory and Australian Capital Territory, only 19 of 1,095 cases were in these jurisdictions.

As expected, mean incidence of candidemia was higher in university hospitals and university-affiliated hospitals, which have a higher proportion of patients at risk for candidemia (0.22/1,000 separations each) than private hospitals (0.1/1,000 separations). Since similar clinical management practices are used throughout Australia, jurisdictional differences are likely caused by different exposures to risk factors for infection. The highest incidences were observed in jurisdictions with organ transplantation, burn, and critical care centers.

Concomitant conditions and risk factors associated with candidemia were similar to those previously described (*11**,**25*), but pancreatitis (*26*) and HIV infection (8%–10% of cases in the United States) (*7**,**11*) were rare, and only 20% of episodes were associated with an ICU (33%–40% elsewhere) (*7**,**13**,**25*). We observed approximately equal numbers of cases in hematologic and solid tumor cancer patients. However, in previous studies, solid tumor patients were more common (*6**,**11**,**25*). Whether the prevalence of candidemia is increasing in the setting of chemotherapy for hematologic malignancy, despite use of antifungal prophylaxis in selected patients, is the subject of ongoing study.

Australian hospital statistics showed a 4% increase in community-based healthcare from 2000 through 2003 (*27*). Using population-based surveillance, we identified ≈20% of candidemia episodes that would not have been captured by nosocomial surveillance. This proportion is higher than in Europe (6%–10%) (*12**,**13*), but lower than in the United States (20%–28%) (*7**,**11*). However, these proportions are not directly comparable since previous studies have used different criteria to define outpatient-acquired episodes. Although most studies required blood cultures to be positive <48 hours of patient admission (*1**,**13*), others have used <72 hours (*28*) or <24 hours (*7**,**11*). If one considers the likely incubation period of candidemia, we suggest that 48 hours is appropriate. Furthermore, previous studies have not distinguished between CA and OHCA infections (*7**,**11**,**13**,**28*).

Using a national standard classification for bloodstream infections (*17*), we identified differences and similarities between IHCA candidemia and candidemia acquired outside a hospital and between OHCA and CA infections. Most (>60%) outpatient cases were healthcare associated. These cases resembled IHCA candidemia because cancer and other chronic diseases were common concomitant conditions and established risk factors were often present, although less frequently, than in IHCA infection; they differed from CA candidemia in instances in which IVDU was a risk factor. Thus, emergence of candidemia outside the hospital setting is likely due to the shift in persons with iatrogenic risk factors, such as chemotherapy and intravenous antimicrobial drug therapy, increasing management of more serious conditions outside the hospital, and implementation of early hospital discharge. Home-based therapies have been implicated in at least 1 outbreak of outpatient candidemia (*29*).

Compared with IHCA infection, the 30-day death rate and duration of hospital stay were lower in OHCA cases. Candidemia may have been more severe in the IHCA group because sepsis was initially identified in a higher proportion of these patients than in OHCA patients and was an independent predictor of death. Concomitant bacteremia was more common in IHCA patients. However, since no control group was studied, the difference may be explained by other characteristics of hospitalized patients compared with outpatients. Clinical outcomes of OHCA and CA candidemia were similar, although sepsis was present at diagnosis in a higher proportion of OHCA patients. Candidemia should be included in the differential diagnosis of patients with appropriate risk factors and sepsis who are admitted to emergency departments or in other healthcare settings.

Species distribution varied by jurisdiction, healthcare setting, age, and hospital service. In South Australia, *C*. *albicans* was less common and *C*. *parapsilosis* was more prevalent. This finding was not explained by differences in age distribution or proportions of IHCA and OHCA infections between jurisdictions. Overall, *C*. *parapsilosis* candidemia was more prevalent in outpatients, many of whom had VADs in situ, and in neonates, which is consistent with previous studies (*7**,**11*). Given the reduced susceptibility of *C*. *glabrata* to azole drugs (*30*), our observations that *C*. *glabrata* candidemia occurs more often in patients without hematologic malignancy and that these infections increased during the study provide useful data. In other studies, *C*. *glabrata* was more common in older persons (*31*) and patients with hematologic cancer (*24*).

The high overall death rate, albeit lower than reported elsewhere (35%–44%) (*7**,**12**,**13*), is another reminder of the role of candidemia in healthcare settings. The highest case-fatality rate was observed in the most vulnerable patients (elderly, ICU patients, and those who recently had medical interventions). Sepsis syndrome and failure to institute treatment with antifungal drugs (the latter occurred mainly in preterminal hematology patients) were independent predictors of death. Although removal of VADs did not independently protect against death, data from univariate analysis (Table 3) support current recommendations to remove VADs when candidemia is detected (*32*).

In conclusion, although candidemia is primarily a healthcare-associated entity in patients with established risk factors, many of these patients are observed in the outpatient setting. OHCA infections have characteristics intermediate between those of IHCA and CA infections. We propose that cases of candidemia be categorized as IHCA, OHCA, and CA, with the term CA reserved for those episodes occurring <48 hours of hospital admission and that do not meet criteria for healthcare-associated infections. Further study of secular trends and characteristics of candidemia acquired outside hospitals with standardized definitions is warranted. Surveillance is needed to track trends of this serious infection and provide guidelines for antifungal prophylaxis, treatment, and infection control strategies.
